# Successive and automated stable isotope analysis of CO_2_, CH_4_ and N_2_O paving the way for unmanned aerial vehicle‐based sampling

**DOI:** 10.1002/rcm.8929

**Published:** 2020-09-23

**Authors:** Simon Leitner, Rebecca Hood‐Nowotny, Andrea Watzinger

**Affiliations:** ^1^ University of Natural Resources and Life Sciences Vienna Institute of Soil Research Konrad‐Lorenz‐Straße 24 Tulln 3430 Austria

## Abstract

**Rationale:**

Measurement of greenhouse gas (GHG) concentrations and isotopic compositions in the atmosphere is a valuable tool for predicting their sources and sinks, and ultimately how they affect Earth's climate. Easy access to unmanned aerial vehicles (UAVs) has opened up new opportunities for remote gas sampling and provides logistical and economic opportunities to improve GHG measurements.

**Methods:**

This study presents synchronized gas chromatography/isotope ratio mass spectrometry (GC/IRMS) methods for the analysis of atmospheric gas samples (20‐mL  glass vessels) to determine the stable isotope ratios and concentrations of CO_2_, CH_4_ and N_2_O. To our knowledge there is no comprehensive GC/IRMS setup for successive measurement of CO_2_, CH_4_ and N_2_O analysis meshed with a UAV‐based sampling system. The systems were built using off‐the‐shelf instruments augmented with minor modifications.

**Results:**

The precision of working gas standards achieved for δ^13^C and δ^18^O values of CO_2_ was 0.2‰ and 0.3‰, respectively. The mid‐term precision for δ^13^C and δ^15^N values of CH_4_ and N_2_O working gas standards was 0.4‰ and 0.3‰, respectively. Injection quantities of working gas standards indicated a relative standard deviation of 1%, 5% and 5% for CO_2_, CH_4_ and N_2_O, respectively. Measurements of atmospheric air samples demonstrated a standard deviation of 0.3‰ and 0.4‰ for the δ^13^C and δ^18^O values, respectively, of CO_2_, 0.5‰ for the δ^13^C value of CH_4_ and 0.3‰ for the δ^15^N value of N_2_O.

**Conclusions:**

Results from internal calibration and field sample analysis, as well as comparisons with similar measurement techniques, suggest that the method is applicable for the stable isotope analysis of these three important GHGs. In contrast to previously reported findings, the presented method enables successive analysis of all three GHGs from a single ambient atmospheric gas sample.

## 1 INTRODUCTION

There is an increased awareness of the anthropogenic impact on climate change. Identifying the sources and sinks of greenhouse gases (GHGs) and monitoring their atmospheric abundance^1^ are essential in managing the required global GHG emission reductions to achieve the 1.5°C target^2^ or to establish pathways to zero emissions.^3^ GHG monitoring is a valuable input to facilitate technology improvement, leading to more efficient resource utilization. The most important GHGs are carbon dioxide (CO_2_), methane (CH_4_) and nitrous oxide (N_2_O). For 2018 the global mean concentrations of CO_2_, CH_4_ and N_2_O were 407.8 ± 0.1 μmol mol^−1^, 1869 ± 2 nmol mol^−1^ and 331.1 ± 0.1 nmol mol^−1^, respectively.^4^ In light of their low absolute concentrations, instrumentation has to be used that will ensure accurate and precise measurements. In addition to the compound concentration, the stable isotopic composition enables identification of GHG sources and sinks.^5^, ^6^, ^7^, ^8^, ^9^ However, the inclusion of isotopic analysis requires different considerations and an appropriate analyzer, such as an isotope ratio mass spectrometer, or optical devices based on tunable diode laser adsorption spectroscopy or Fourier transform infrared spectroscopy.^10^ While optical devices are customized to analyze specific compounds only (e.g. CO_2_, H_2_O or N_2_O), isotope ratio mass spectrometry (IRMS) can be used to measure a multitude of compounds in addition to GHGs. The key process in efficient and accurate IRMS is sample preparation, which encompasses the various steps from specifically designed sample collection and manual sample preparation, through to introduction of the gases into the device and treatment options for specific gas separation.

Most IRMS systems use gas chromatography (GC) to separate and isolate gas compounds of similar physical and chemical behavior (e.g. CO_2_ and N_2_O). Furthermore, pre‐concentration steps connected in series with GC are often needed, if injection volumes (e.g. 20 mL to 2.5 L)^11^, ^12^ yield compound amounts (e.g. of CH_4_, N_2_O) below IRMS detection limits, which are in the range of hundreds of picomoles to nanomoles. A common pre‐concentration approach is the use of cryogenic traps, filled with adsorbent material to trap CH_4_ or N_2_O while other residual compounds are vented away. In this setup the entire gas contents of the vessel, whatever the volume, are purged out though the trapping system with a carrier gas such as helium. Pre‐concentrated CH_4_ held on the trap can then be released and oxidized to CO_2_, while trapped pre‐concentrated N_2_O requires no chemical transformation for detection. Both gases then undergo similar preparation to that for atmospheric CO_2_ measurements. That is, compounds are focused and separated from any residuals using GC and transferred to the isotope ratio mass spectrometer, which measures the intensity of *m*/*z* 44, 45 and 46 to calculate the stable isotope ratios of carbon, oxygen or nitrogen of sampled CO_2_, CH_4_ or N_2_O.

There are already numerous methods available to measure atmospheric samples for the concentration and isotopic composition of CO_2_, CH_4_ and N_2_O.^11^, ^12^, ^13^, ^14^, ^15^, ^16^, ^17^, ^18^ While the majority of published methods focus on one of the three GHGs only, the aim of the study reported here was to enable the measurement of all three gases from identical sample vessels in a single‐push measurement approach. Specifically, taking into consideration the compatibility of the sampling approach with unmanned aerial vehicle (UAV)‐based sampling systems, the key for success was the identification of appropriate sample vessels fitting the requirements of both the sampling system and the measurement setup. To our knowledge there is no comprehensive GC/IRMS setup for the successive measurement of CO_2_, CH_4_ and N_2_O meshed with a UAV‐based sampling system.

This paper presents a method for automated GC/IRMS based on simultaneous and/or successive measurement of atmospheric CO_2_, CH_4_ and N_2_O provided by a single air sample. Therefore, sample vessels have been identified fitting the requirements of UAV‐based sampling systems and GC/IRMS instrumentation. Such a UAV‐based sampling system was also designed and tested, but will be presented elsewhere. In accordance with Schauer et al,^17^ the aim was to employ off‐the‐shelf instruments needing minor modification only, so that the presented methods can be considered by the scientific community as an alternative to specially designed instruments. It should also be kept in mind that, after adjusting the current measurement system, it is still ready to use for its ordinary purpose of analyzing a large variety of other gaseous and volatile compounds.

The presented methods were customized to the current abundance status of CO_2_, CH_4_ and N_2_O in the atmosphere. The detection range for atmospheric CO_2_ and CH_4_ was established at 372 to 944 μmol mol^−1^ and 1.7 to 5.0 μmol mol^−1^, respectively.^19^ Appropriate limits of determination for tracing atmospheric gases are recommended at 100 μmol mol^−1^ for CO_2_ and 500 nmol mol^−1^ for CH_4_ ^20^ in Schuyler and Guzman.^21^ For N_2_O, a limit of determination of 300 nmol mol^−1^ was the target to guarantee the measurement of current atmospheric global mean abundance.

## 2 MATERIALS AND METHODS

In the process of setting up the methods described below, there have been many process iterations with several discarded options, in terms of sample vial specifications, sample transfer, GC columns, adsorbents and cryogenic trapping. These are summarized in the supporting information.

### 2.1 Sample vial preparation

Air was sampled in 20‐mL  headspace vials (La‐pha‐pack GmbH, Langerwehe, Germany) sealed with grey butyl‐PTFE‐lined septa (DWK Life Sciences GmbH, Mainz, Germany) and aluminum crimp caps.^22^, ^23^ The vials were pre‐conditioned by flushing with helium or synthetic air for 1 min at an inlet pressure of 2 bar before evacuation for 1 min with a rotary vane pump (e.g. E2M‐1.5, Edwards Ltd, Burgess Hill, UK) to a final pressure of approx. 0.05 Pa. Flushing was performed using two G26 51 mm Luer‐lock side‐bore needles (Hamilton Bonaduz AG, Bonaduz, Switzerland) while evacuation used a single needle only. Pre‐conditioned or already filled vials can be stored for several weeks before measurement, having shown adequate tightness in internal tests and according to the literature.^23^, ^24^

### 2.2 Sampling of CO_2_ via direct injection to GC/C‐HTC/IRMS

**FIGURE 1:**
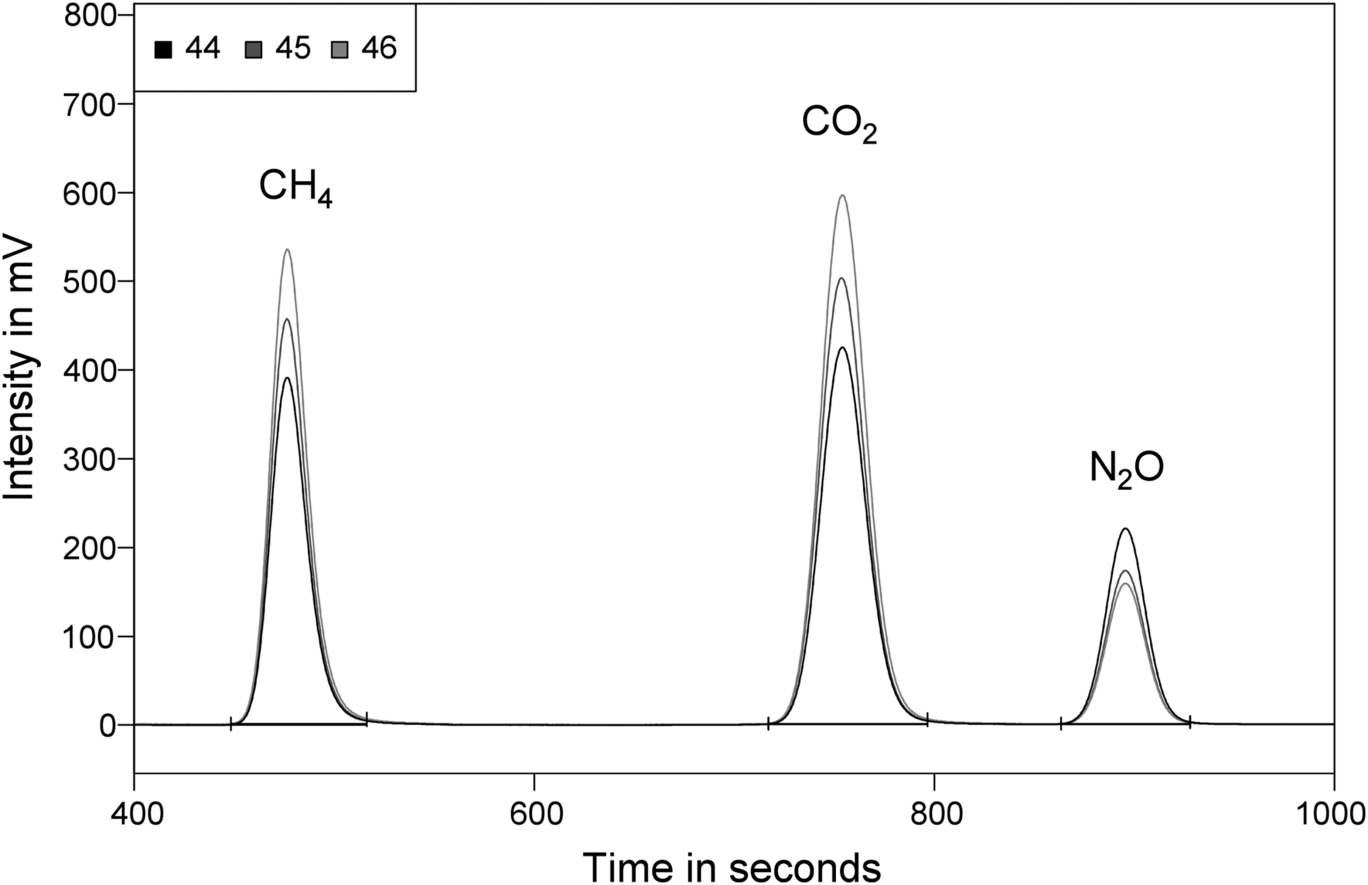
Chromatographic separation of CH_4_, CO_2_ and N_2_O via direct injection into GC/C‐HTC/IRMS. Peak areas represent 4.6, 7.0 and 4.7 nmol on GC column

This method sought to achieve rapid accurate automated measurement, using a sequential sampling procedure for CO_2_. Up to 64 samples can be loaded to the autosampler (Combi PAL, CTC Analytics AG, Zwingen, Switzerland) and an aliquot sample volume of 300 μL was transferred by the autosampler to a split/splitless injection port of the gas chromatograph (Trace GC Ultra, Thermo Fisher Scientific S.p.a., Rodano, Italy). A 1‐mL  syringe (1.0 mL HLT PTFE sealed G23, Innovative Laborsysteme GmbH, Stützerbach, Germany) maintained at room temperature was initially flushed with helium 5.0 taking two filling strokes before injecting the sample at 50 μL s^−1^. The injector was maintained at 120°C housing a 3 mm inner diameter splitless liner (LNR TQ CE, Trajan Scientific and Medical, Ringwood, Australia). Sample compounds were separated and focused on a packed column (ShinCarbonST 80/100 mesh, 2 m × 1 mm inner diameter, Restek Corporation ordered from BGB Analytik AG, Rheinfelden, Switzerland)^25^ with a temperature program starting at 40°C, and increased at 20°C min^−1^ to 150°C where it was held for 5 min before being increased at 50°C min^−1^ to the final temperature of 180°C. Furthermore, it is important to note that the splitless time of the injector was 1 min and the septum purge was stopped for 1 min. The He carrier gas provided a constant pressure of 150 kPa. This procedure enables a sufficient separation of CO_2_, but it can be altered if CH_4_ and/or N_2_O are present in concentrations similar to that of CO_2_ (Figure 1).

The temperature program was then adjusted as follows. Starting at 40°C, the temperature was held for 1 min before being increased at 10°C min^−1^ to 110°C where it was held for 5 min before being increased to 180°C at 20°C min^−1^. In addition, the carrier gas pressure was maintained at 150 kPa for 8.4 min and increased to 180 kPa at 50 kPa s^−1^ for the remaining time. Thereby CH_4_, CO_2_ and N_2_O were separated and focused properly and the issue of interfering N_2_ with CH_4_ and H_2_O with CO_2_ and N_2_O was overcome. After being eluted, CH_4_ was passed through a Cu/Ni combustion/reduction reactor (1000°C) and oxidized to CO_2_, while air–CO_2_ and air–N_2_O were passed through an empty AlO_3_ tube maintained at 200°C (designed as a high‐temperature‐conversion (HTC) reactor operating at temperatures above 1000°C) to preserve their chemical state. All three analytes were successively transferred via a universal continuous flow gas interface (ConFlo IV, Thermo Fisher Scientific GmbH, Bremen, Germany) to the isotope ratio mass spectrometer (Delta V Advantage, Thermo Fisher Scientific GmbH) measuring the ion currents at *m*/*z* 44, 45 and 46. This approach of direct injection was shown to be consistent for the measurement of CO_2_, CH_4_ and N_2_O at levels above 250 μmol mol^−1^ in air.

### 2.3 Sampling of CH_4_ and N_2_O via purge and trap GC/C‐HTC/IRMS

**FIGURE 2:**
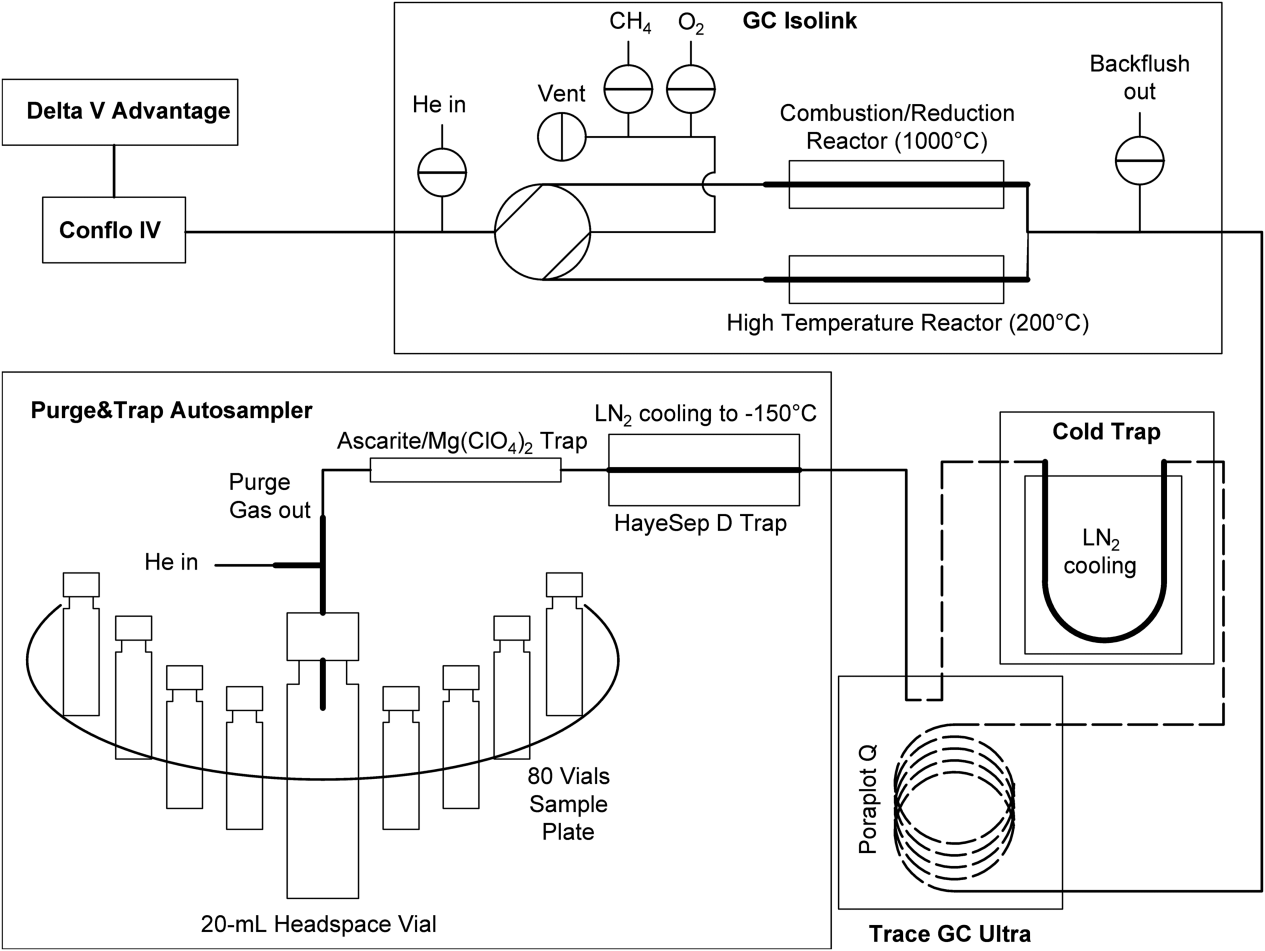
Illustration of measurement procedure for CH_4_ and N_2_O in the example of purge and trap autosampling drawn in the CH_4_ flow path

For measurement of CH_4_ or N_2_O, concentration range of 0.3 to 3.5 μmol mol^−1^ in air, the samples were switched to a purge and trap autosampler (VSP 4000, Envea™, SWR Engineering GmbH, Schliengen, Germany). The general procedure is illustrated in Figure 2.

The autosampler was modified such that the cryo‐trap (1/16″ outer diameter, 0.8 mm inner diameter × 136 mm glass‐lined tube) was filled along 21 mm with HayeSep D mesh 80/100 (HayeSeparations Inc., Bandera, TX, USA) following Miller et al.^12^ Water and CO_2_ were trapped in front of the cryo‐trap using a CO_2_/water trap (100 mm × 1/2″ outer diameter glass tube, IVA Analysetechnik GmbH, Meerbusch, Germany) filled with an equal mixture of Ascarite and anhydrous magnesium perchlorate (Mg(ClO_4_)_2_). In order to enable the use of identical crimp vials to those used for direct injection a specially designed needle plate was used. Similar to the gas‐bench needle approach, a Pencan Paed G22 0.6 mm outer diameter needle (B. Braun Austria GmbH, Maria Enzersdorf, Austria) was cut at 100 mm, to serve as the purge gas outlet, and was inserted into a G19 1.0 mm outer diameter Luer‐lock needle (Hamilton Bonaduz AG), cut at 50 mm, working as helium inlet, protruding by 3 mm. The G19 needle terminated inside a 1/16″ stainless steel T‐piece (Swagelok, ordered from AA‐Solutions GmbH, Wiener Neudorf, Austria), sealed with a drilled‐out 0.25 mm inner diameter SilTite ferrule (Fisher Scientific Austria, Vienna, Austria) connected to the helium inlet (1/16″ stainless steel tube) and a 1/16″ stainless steel connector (Swagelok, ordered from AA‐Solutions GmbH) leading to the G22 needle, sealed with three drilled‐out 1/16″ × 0.5 mm inner diameter graphite/vespel ferrules (Restek Corporation ordered from BGB Analytik AG) to a 1/16″ stainless steel tube purge gas outlet.

**FIGURE 3:**
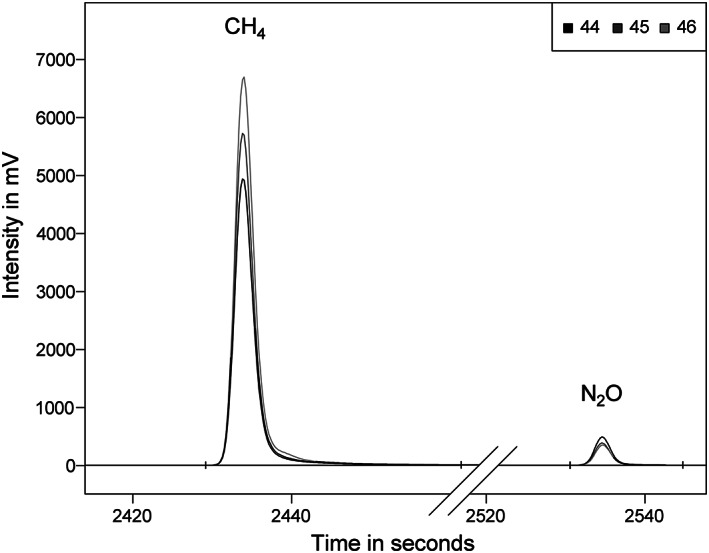
Chromatographic separation of CH_4_ and N_2_O with the injection of three ambient atmospheric air samples (sample volume of 61.5 mL) with the purge and trap autosampler

Vials were each purged with helium 5.0 for 10 min at a flow rate of 20 mL min^−1^, while the cryo‐trap was maintained at −150°C^26^ by cooling with liquid nitrogen (LN_2_). Due to the limit of determination and calibration range of the presented method, measurement of atmospheric N_2_O required a sample volume of three sample vials (61.5‐mL ), as shown in Figure 3, while the volume of a single vial (20.5‐mL ) was sufficient for the analysis of CH_4_. Therefore, the software of the VSP 4000 had to be modified to enable the trapping of multiple vial volumes in a single measurement keeping the cryo‐trap at −150°C while switching the purge procedure from vial to vial. To finalize the purge process, the cryo‐trap was heated to 120°C at 50°C s^−1^, desorbing the trap and transferring gaseous compounds for 120 s at a head pressure of 700 hPa. The VSP 4000 was connected via a temperature programmed transfer line (200°C) enabling an on‐column injection. In front of the GC column (Poraplot Q, 30 m × 0.32 mm inner diameter, 10 μm film, Agilent Technologies Austria GmbH, Vienna, Austria) a guard column was installed (3‐m  piece of Poraplot Q, 0.32 mm inner diameter, 10 μm film), which guided the transferred compounds from the transfer line outlet inside the gas chromatograph through a 1/16″ stainless steel tube (U‐shaped, length of 50 cm) mounted to the GC‐Isolink Cold Trap Option (Thermo Fisher Scientific GmbH) piston cantilever. The stainless steel tube was immersed in a 3‐L  LN_2_ Dewar flask to separate CH_4_ from residual N_2_ and O_2_. Subsequently the guard column was linked back into the gas chromatograph and connected to the GC column. The cold trap was activated 30 s before the cryo‐trap was heated up and was kept immersed in LN_2_ during and after the transfer time for a total of 240 s after cryo‐trap desorption started. The GC temperature gradient was programmed to start at the beginning of the purge and trap transfer time keeping a temperature of 35°C for 8.7 min before it was increased to 120°C at 50°C min^−1^ to reduce the amount of residual water inside the Poraplot Q. The outlet of the GC column was linked to a combustion reactor and a HTC reactor. While the combustion reactor was used for CH_4_ conversion the HTC reactor was set to 200°C to enable the direct measurement of N_2_O via the signals at *m*/*z* 44, 45 and 46. In order to enable simultaneous analysis of CH_4_ and N_2_O in a single run, the gas flow to the isotope ratio mass spectrometer was switched from combustion mode to HTC mode at 265 s after the cold trap had been lifted. Switching the reactor also required switching the gas configuration in the ISODAT software (Thermo Fisher Scientific GmbH) from CO_2_ to N_2_O.

### 2.4 Referencing and calibration of CO_2_, CH_4_ and N_2_O

The isotopic compositions of CO_2_, CH_4_ and N_2_O are reported in δ‐notation (‰) and were referenced against laboratory working standard gases (CO_2_, N_2_O) which have been calibrated relative to the international standard Vienna Peedee Belemnite (VPDB) for δ^13^C and δ^18^O values^27^, ^28^ and AIR‐N_2_ for the bulk δ^15^N value using internationally distributed isotopic reference materials. The δ‐values were calculated as:

$$\delta^{13}C=\frac{R_{P}}{R_{\mathrm{Std}}}-1\;\delta^{18}O=\frac{R_{P}}{R_{\mathrm{Std}}}-1\;\delta^{15}N_{\text{bulk}}=\frac{R_{P}}{R_{\mathrm{Std}}}-1$$

where *R* is the ratio of the abundance of ^13^C to ^12^C, ^18^O to ^16^O and ^15^N to ^14^N of a sample (P) and a measurement standard (Std).^29^

A CO_2_ working gas (CO_2_ 4.8 F50, Messer Austria GmbH, Gumpoldskirchen, Austria) and a CH_4_ working gas (CH_4_ 4.5 F10, Messer Austria GmbH) were calibrated using the above described direct injection method using two certified CO_2_ gas standards (ISO‐TOP, Messer Austria GmbH) with a δ^13^C value of −6.7 ± 0.2‰ and −39.0 ± 0.2‰ and a δ^18^O value of −7.8 ± 0.2‰ and −20.4 ± 0.2‰ vs VPDB. The N_2_O working gas (N_2_O UHP F10, Messer Austria GmbH) was converted into N_2_ using the GC‐Isolink combustion/reduction reactor and was then measured against an N_2_ working gas, used as the measurement reference gas (N_2_ 5.0, F10, Messer Austria GmbH), which had been calibrated using elemental analyzer/IRMS (Flash 2000–ConFlo IV–DeltaV Advantage, Thermo Fisher Scientific GmbH, Cambridge, UK) using the international standard materials IAEA‐N‐1 (0.4 ± 0.1‰),^30^ IAEA‐NO‐3 (4.7 ± 0.1‰)^31^ and IAEA‐600 (0.9 ± 0.1‰).^32^ In addition, pure N_2_ and N_2_O working gases were manually injected into the elemental analyzer and using the N_2_ working gas as the measurement reference gas for IRMS to determine the bulk δ^15^N value of the N_2_O working gas. The determination of the δ^13^C and δ^18^O value of CO_2_ and the δ^13^C value of CH_4_ as well as the bulk δ^15^N value of N_2_O followed the evaluation procedure of Paul and Skrzypek.^33^ The working gases indicated an isotopic composition and uncertainty of −29.5 ± 0.1‰ for the δ^13^C value and 1.2 ± 0.1‰ for the δ^18^O value of CO_2_ (*n* = 6), −40.3 ± 0.2‰ (*n* = 38) for the δ^13^C value of CH_4_ and −1.2 ± 0.1‰ (*n* = 4) for the bulk δ^15^N value of N_2_O.

Each reported sample run implies the use of one of the three working standard gases (CO_2_, CH_4_, N_2_O) as a measurement reference gas injected as a working gas standard for isotopic evaluation and compound quantification. For preparing working gas standards, CO_2_ was added pure, while CH_4_ and N_2_O were first diluted to a specific concentration range using pure gas addition to a glass gas mouse flushed and filled to laboratory air pressure with synthetic air (EPA‐quality F50, Messer Austria GmbH). Samples and working gas standards were prepared under identical treatment,^34^ equilibrating the vials under pressure with sample gas (atmospheric air) or synthetic air using a 30‐mL  all‐glass syringe (Fortuna® Optima®, Poulten & Graf GmbH, Wertheim, Germany) with a 51 mm G26 side‐bore Luer‐lock needle (Hamilton Bonaduz AG) installed using three filling strokes of sample gas in advance. Vials filled with synthetic air were used as working gas standards by adding pure or diluted working gases (7–15 μL). Samples were calibrated on a daily basis. The calibration range of CO_2_ spanned from 340 to 730 μmol mol^−1^ (315–675 nmol on GC column). Working gas standards from diluted CH_4_ ranged from 0.7 to 7.1 μmol mol^−1^ (0.57–5.82 nmol on GC column) and for diluted N_2_O from 0.3 to 2.9 μmol mol^−1^ (0.26–2.56 nmol on GC column).

## 3 RESULTS AND DISCUSSION

### 3.1 Internal calibration of CO_2_, CH_4_, N_2_O

#### 3.1.1 CO_2_

Working gas standards of three nominal CO_2_ concentration levels (341, 488 and 732 μmol mol^−1^) were prepared in duplicate. Working gas standard vials were measured five times in succession spread over the sequence. The measured data were processed as follows. In order to calculate the concentration, the AreaAll value (V s) was correlated with the vial's respective nominal concentration by linear regression, resulting in a correlation coefficient (*R* ^2^) of 1.0 and *p* < 2 × 10^−16^. Measurements of blanks, containing synthetic air only, showed an amplitude of mass 44 of 18 to 30 mV for the first to the last injection and did not affect the raw delta values of working gas standards or air samples. The raw delta values were checked and corrected to the mass spectrometer signal response (ion source linearity), measurement reference gas (CO_2_) disparities of the backflush and straight mode operation, and offsets between the nominal and measured delta values of the CO_2_ working gas. Corrections for signal response linearity were calculated by a quadratic polynomial (*R* ^2^ > 0.98) that provided a better fit than a linear function^35^ (beam intensity of 300 to 6500 mV), resulting in corrections of 0.1 to 0.4‰ for δ^13^C values and 0.0 to −0.2‰ for δ^18^O values for an amplitude of mass 44 of 260 to 700 mV. Measurement reference gas disparities in the backflush and straight mode operation did not affect the calculated isotope ratios. The offset between the nominal and measured delta value of the CO_2_ working gas was 0.2 to 0.4‰ for δ^13^C values and −0.5 to 0.1‰ for δ^18^O values, both at decreasing amplitude of mass 44.

The corrected values were checked for outliers using the Grubbs test^36^ (setting a significance level of 5%). Therefore, values were grouped by nominal concentration to obtain three groups of ten values each. A single outlier was detected, which was discarded. The overall arithmetic mean value and standard deviation (SD) of the δ^13^C and δ^18^O values (*n* = 29) of the CO_2_ working gas standards were −4.3 ± 0.3‰ and −3.1 ± 0.3‰, respectively, with a maximum SD of 10 μmol mol^−1^ over the entire concentration range. A confidence interval of twice the SD (95%) for δ^13^C and δ^18^O values covered 93% and 90% of the values, respectively. Thereby, the overall arithmetic mean values slightly changed to −4.4‰ and −3.1‰ while the SD for δ^13^C and δ^18^O values dropped to 0.2‰ and 0.3‰, respectively. The results are shown in Figure S1 (supporting information) for individual data points of measured working gas standards and presented in Table 1, giving a summary of grouped data points.

**TABLE 1:** Summary of the internal calibration of the CO_2_ method. Working gas standards were prepared at three nominal concentration levels as duplicates measured five times each (*n*_tot_ = 30). *n* is the number of injections with outliers discarded due to the Grubbs test and within a 95% confidence interval of the δ^13^C and δ^18^O values (separated by a slash). Mean values are arithmetic means of *n* with their respective standard deviation (SD) and concentration (*c*)

Nominal *c* (μmol mol^−1^)	*n*	Mean *c* (μmol mol^−1^) ± SD	Mean δ^13^C (‰) ± SD	Mean δ^18^O (‰) ± SD
341	10/8	348 ± 4	−4.4 ± 0.3	−3.0 ± 0.2
488	8/9	488 ± 2	−4.5 ± 0.2	−3.0 ± 0.4
732	10/10	729 ± 10	−4.3 ± 0.2	−3.1 ± 0.2

#### 3.1.2 CH_4_ and N_2_O

Internal calibration of CH_4_ and N_2_O covered the range 0.7–2.8 and 0.8–2.9 μmol mol^−1^, respectively. The purge and trap method, which was designed to measure ambient atmospheric CH_4_ and N_2_O, was tested by repeated preparation and measurement of atmospheric samples and working gas standards in six individual sequences across a period of two weeks. Samples and standards were measured from single and multiple vials with standard vials always containing both working standard gases (CH_4_ and N_2_O). The measurement results of the working gas standards were grouped by sequence and nominal concentration to calculate measured concentrations by linear regression of their AreaAll (V s) and nominal concentration. All values were inside a 95% confidence interval. The regression coefficients settled between 0.97 and 1.0, and the *p*‐values were kept below 4.5 × 10^−6^ for both CH_4_ and N_2_O. Delta value corrections due to ion source linearity and measurement reference gas (CO_2_, N_2_O) offset between backflush and straight mode operation could be neglected due to their observed insignificant contribution. While there were no peaks at the retention time of N_2_O in blank measurements, there was a small peak interfering with the CH_4_ peak. These blank peaks were similar in AreaAll and δ^13^C values in measurements of multiple blank vials within each sequence which enabled a straightforward subtraction from calibration gas standards and sample values.^37^

A Grubbs test did not identify any outliers for δ^13^C values of CH_4_ or δ^15^N values of N_2_O. The overall arithmetic mean value and SD of the δ^13^C and δ^15^N values was −40.2 ± 0.4‰ and 4.6 ± 0.4‰, respectively. Excluding values outside a 95% confidence interval kept 94% and 96% of the values of each dataset, which are presented in Figure S2 (supporting information). The calculated mean values remained unchanged, while the SDs decreased to 0.4‰ and 0.3‰ for CH_4_ and N_2_O, respectively. A summary of the results grouped by the nominal compound amount is presented in Table 2. It should be noted that mean values were calculated by merging day‐to‐day measurements which gives the value information about the medium‐term (period of two weeks) stability of the method.

**TABLE 2:** Results of CH_4_ and N_2_O working gas standard measurements: *n*_tot_ represents the total number of measurements, and *n* is the number of measurements inside a 95% confidence interval. Nominal concentrations are indicated by *c*_nom_ in μmol mol^−1^ and measured concentrations (*c*_mean_) are calculated as arithmetic means of μmol mol^−1^ with their standard deviation (SD). Delta values are given as arithmetic means in ‰ with their SD. For N_2_O, the column *S*_vol_ indicates the total sample volume, which can represent either a single vial volume (20.5 mL) or the volume of three vials (61.5 mL)

CH_4_						N_2_O						
*c*_nom_	*n*_tot_	*n*	*c*_mean_ ± SD	RSD (%)	δ^13^C ± SD	*c*_nom_	*S*_vol_	*n*_tot_	*n*	*c*_mean_ ± SD	RSD (%)	δ^15^N ± SD
0.68	3	3	0.69 ± 0.04	6	−40.3 ± 0.3	0.83	20.5	8	7	0.75 ± 0.10	13	4.6 ± 0.3
0.88	8	7	0.86 ± 0.04	5	−40.3 ± 0.4	0.88	20.5	12	11	0.90 ± 0.05	6	4.6 ± 0.4
1.17	7	7	1.18 ± 0.05	4	−40.2 ± 0.5	1.17	20.5	7	7	1.15 ± 0.02	2	4.6 ± 0.1
1.37	4	4	1.37 ± 0.11	8	−40.1 ± 0.5	1.76	20.5	11	11	1.77 ± 0.09	5	4.6 ± 0.3
1.76	6	6	1.85 ± 0.11	6	−40.3 ± 0.4	2.63	61.5	3	3	2.65 ± 0.01	0	4.6 ± 0.2
2.10	6	6	2.12 ± 0.10	5	−40.3 ± 0.3	2.78	20.5	8	8	2.42 ± 0.20	8	4.6 ± 0.3
2.78	12	10	2.84 ± 0.16	6	−40.2 ± 0.3	2.93	20.5	2	2	3.01 ± 0.00	0	4.5 ± 0.1
2.93	2	2	2.93 ± 0.01	0	−40.2 ± 0.1	3.37	61.6	6	6	3.71 ± 0.23	6	4.6 ± 0.2

Measurements of ambient atmospheric air samples indicated a necessary sample volume of 61.5 mL (volume of three vials). In order to save consumables and device run time, working gas standards were measured from single vial volumes. To ensure accurate quantification when standards and samples were measured from different vial volumes, the N_2_O concentrations from single vial volumes and triple vial volumes were compared. As is evident from Table 2 for the nominal concentration of 0.88 and 1.17 μmol mol^−1^ (from single vial volume, 20.5 mL) and 2.63 and 3.37 μmol mol^−1^ (from triple vial volume, 61.5 mL), the delta values and concentrations showed consistency.

### 3.2 Analysis of atmospheric air

#### 3.2.1 CO_2_

**FIGURE 4:**
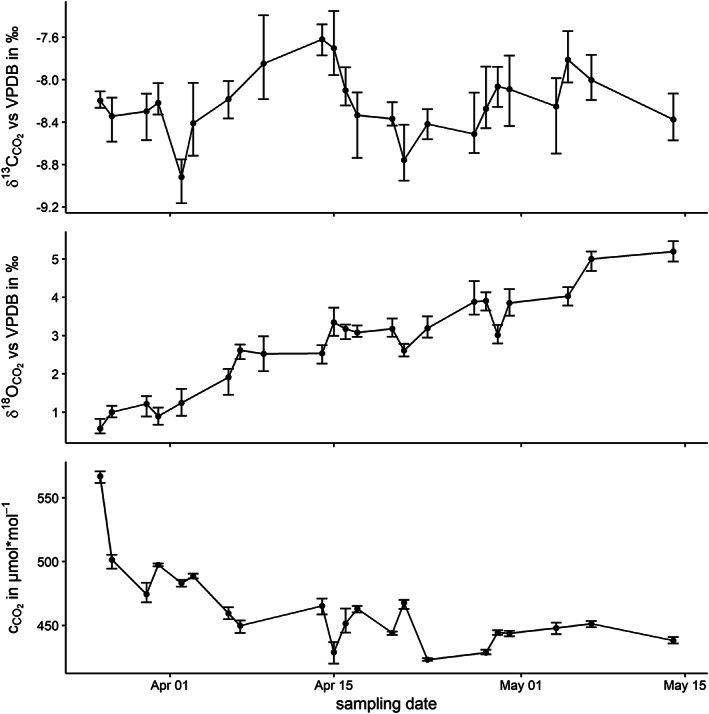
Progress of CO_2_ concentration and isotopic composition in April to May 2020. Mean values are displayed as points with the minimum and maximum value from consecutive injections indicated as an error bar

The method for CO_2_ measurements of atmospheric samples was tested by consecutive sampling of atmospheric air outside the institute building (WGS84 coordinates; E: 16.067816 N: 48.320476), located in a suburb, and surrounded by agricultural land at a height of 3.4 m above ground, in March to May 2020. Figure 4 shows the δ^13^C and δ^18^O values and respective concentrations of CO_2_ of the measured atmospheric samples. Each vial was measured four times and the results were filtered using a Gibbs test to detect outliers and a 95% confidence interval. The residual values comprised 97%, 91% and 87% of the initial datasets with a maximum SD of 0.3‰ and 0.4‰ for δ^13^C and δ^18^O values, respectively, and 9 μmol mol^−1^ for concentrations. Figure 4 presents mean values with error bars indicating minima and maxima of filtered data points. The values of δ^13^C aligned with reported values of the three closest ESRL Global Monitoring Laboratory observation sites in Germany (Hohenpreissenberg – HPB; Ochsenkopf – OXK) and Hungary (Hegyhatsal – HUN).^38^ It was speculated that the trend of ^18^O enrichment and the decrease in CO_2_ concentration was driven by atmosphere/leaf diffusion processes and the CO_2_ demand by increasing photosynthetic activity during the sampling period in springtime.^39^

Weather conditions such as temperature and partial pressure affect the volume of sampled air. If atmospheric samples have been stored for several weeks at constant room temperature it is recommended that the vial's pressure should be equilibrated by the addition of synthetic air to reduce the impact of laboratory air CO_2_ and to account for the added volume in the calculation of concentrations.

#### 3.2.2 CH_4_

**FIGURE 5:**
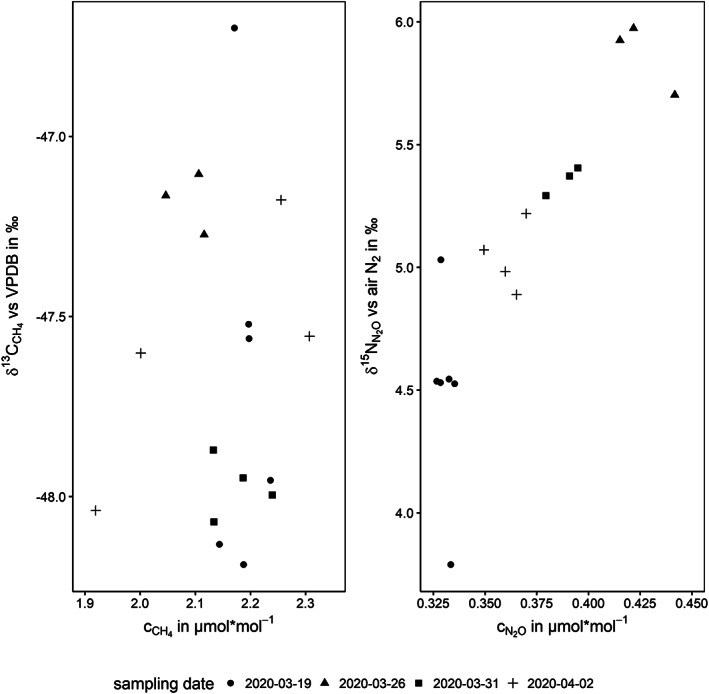
Results of CH_4_ and N_2_O measurements from atmospheric air samples

Sampling of the atmosphere for CH_4_ was carried out in March to April 2020 on four different days to test the repeatability of day‐to‐day measured samples. According to Miller et al,^12^ the expected seasonal variations of δ^13^C values of CH_4_ should be minor on a day‐to‐day basis. The results indeed showed minor variations in the mean values, from −47.2‰ to −48.0‰ over the concentration range of 2.09 to 2.16 μmol mol^−1^ (3 ≤ *n* ≤ 6), as presented in Figure 5. The SDs of the delta values and concentrations were from 0.1‰ to 0.5‰ and 0.03 to 0.19 μmol mol^−1^ with all data points inside a 95% confidence interval.

#### 3.2.3 N_2_O

N_2_O and CH_4_ can be measured using the same procedure but with the need to triple the sample volume for N_2_O (61.5 mL) compared with the CH_4_ evaluation. Atmospheric N_2_O was sampled on the same days as atmospheric CH_4_ and the samples were measured in the same sequences, complemented by working gas standards, which always contained both CH_4_ and N_2_O working gases. The results, presented in Figure 5, indicate natural variations in δ^15^N values (4.5–5.9‰; SD: 0.1–0.4‰; 3 ≤ *n* ≤ 6) and concentrations (0.33–0.43 μmol mol^−1^; SD: 0.00–0.01 μmol mol^−1^) as expected during springtime at a grassland and agriculture surrounded environment.^40^ The depletion in ^15^N compared with the average tropospheric background of 6.55‰ was suspected to result from the impact of soil emissions (δ^15^N values below 0‰) and soil uptake and a probable application of fertilizers (δ^15^N value near 0‰) to agricultural land.^41^, ^42^

## 4 CONCLUSIONS

The method precisions are 0.2‰ and 0.3‰ for the δ^13^C and δ^18^O values of CO_2_, 0.4‰ for the δ^13^C value of CH_4_ and 0.3‰for the δ^15^N value of N_2_O, and cannot reach precisions at the sub‐0.0‰ level as presented by Ehleringer and Cook^43^ for CO_2_, Rice et al^13^ and Miller et al^12^ for CH_4_ or Kaiser and Röckmann^44^ for N_2_O. They are, however, within reach of the precisions obtained^11^, ^45^, ^46^, ^47^ for CH_4_ or N_2_O. Most of these and other publications showing the best precisions use a PreCon‐based system,^14^ which was specially designed for CH_4_ and N_2_O analysis at atmospheric concentrations.

The as yet unsolved weakness of the presented methods relates to the CH_4_ quantification. Despite the observed homogeneity of δ^13^C values obtained by single‐ and triple‐vial volume measurements there was a disproportionate increase in the blank peak. This peak tended to cause overestimation of the CH_4_ concentrations of working gas standards and air samples depending on that peak. Given sufficient yields and appropriate regression coefficients, the measurement of single‐vial volumes is recommended, if both isotopic analysis and quantification of CH_4_ are envisaged. If isotope analysis is the sole objective, the isotope ratios of CH_4_ and N_2_O can be obtained from a single measurement using three sample vials. Moreover, a single sample vial can be used for a preceding measurement of CO_2_ concentration and isotopic ratios. It was therefore shown that successive quantification and stable isotope analysis of all three GHGs in a single ambient atmospheric gas sample can be accomplished by modifying a purge and trap autosampler connected to a GC/C‐HTC/IRMS system. Given that, the envisaged UAV‐based air sampling system can be used to sample the atmosphere for GHGs. Such a system facilitates sampling campaigns at hard‐to‐access areas and enables automated sampling by remote control.

### PEER REVIEW

The peer review history for this article is available at https://publons.com/publon/10.1002/rcm.8929

## Supporting information


**Figure S1:** Data points from CO2 working gas standard measurements. Symbols indicate the injection from each vial. Working gas standards were prepared in duplicate in three nominal concentrations (indicated by dotted vertical lines).
**Figure S2:** Internal calibration of CH4 and N2O working gas, dots and triangles represent sample volumes of 20.5 and 61.5 mL, respectively.
