# Supplementary material for: Successive and automated stable isotope analysis of CO2, CH4 and N2O paving the way for unmanned aerial vehicle‐based sampling
Source: Rapid Commun Mass Spectrom. 2020 Sep 23;34(24):e8929. doi: 10.1002/rcm.8929 (PMC7540016; doi:10.1002/rcm.8929)
Supplement: Supplementary file 1 — Figure S1: Data points from CO2 working gas standard measurements. Symbols indicate the injection from each vial. Working gas standards were prepared in duplicate in three nominal concentrations (indicated by dotted vertical lines). Figure S2: Internal calibration of CH4 and N2O working gas, dots and triangles represent sample volumes of 20.5 and 61.5 mL, respectively. [file RCM-34-e8929-s001.docx]

**Successive and automated stable isotope analysis of CO_2_, CH_4_ and N_2_O paving the way for UAV-based sampling**

Simon Leitner^1^, Rebecca Hood-Nowotny^1^, Andrea Watzinger^1^

^1^ University of Natural Resources and Life Sciences Vienna, Institute of Soil Research, Konrad-Lorenz-Straße 24, 3430 Tulln, Austria

**Supporting Information**

**Lessons learnt from method development**

In order to ease the process of setting up a similar method we would like to share some information about approaches that we tried, but that did not work out. The most crucial part of setting up the method is to identify bottlenecks in fitting your planned setup to your current measurement setup.

In our case finding appropriate sample vessels that could be fitted to either the purge and trap autosampler or the CTC CombiPal was critical. Furthermore, providing sufficient sample volume and sealing quality was most challenging. First, a sample volume of 160 mL ^1^ for the analysis N_2_O was targeted. Based on the notion that sampling of the atmosphere should be done by an UAV-based sampling system, it was planned that the effort of sampling, as well as the space, weight and time required for sampling, should be shortened and that sampling should take place via pressure equilibration of evacuated 20-mL glass vials. In order to reach the required volume of 160 mL using 20-mL vials the purge and trap autosampler software had to be modified to trap the purge gas of multiple vials. The requirement was that the glass vial sealing system must hold under a pressure of approx. 0.05 Pa and must enable sampling via a needle and/or syringe suitable for gases. Side-bore needles with gauge 23 to 26 worked best while red BTO-septa and screw caps did not guarantee sufficient vessel tightness for more than a few hours. Labco exetainer® vials were tested as an alternative to grey-butyl rubber septa and aluminum crimp caps, but they are not available in volumes above 12 mL. The shore (hardness of the rubber) and thickness of the grey-butyl rubber septa required the CombiPal syringe needles to be prevented from bending by gluing an approx. 2 cm piece of plastic tube around the syringe body-to-needle transition and then adjusting the penetration depth. I-LOC® septa with screw caps can be also be fitted to the 23 x 78 mm thread 40-400 glass vial whose specification stated proper sealing, but they could not be integrated into our system due to their high shore value.

The next challenge was to find a suitable GC column which enabled separation of N_2_, CH_4_, CO_2_ and N_2_O when injecting large volumes of air into a gas chromatograph equipped with a regular split/splitless (S/SL) injector and operating at a starting temperature of 35°C or above and a maximum inlet pressure of 250 kPa. Using a single or two-in-tandem Poraplot Q or a Plot Molsieve 5Å column was not successful while the proposed ShinCarbon ST packed column was conclusively the best option. We also tested the injection via a programmed temperature vaporizer injector (PTV) cooled to -50°C with LN_2_, but cannot recommend it. Unfortunately, the ShinCarbon ST column can only be used when injecting CH_4_, CO_2_ and/or N_2_O at concentrations above 250 µL*L^-1^ due to broad peaks at inlet pressures of 250 kPa and below. The S/SL liner and the injection procedure were tested at different nominal volumes of the S/SL liner. Large volume splitless liners worked best and especially at low injection speeds of 50 µL*s^-1^ combined with stopping the septum purge for 1 min, and a splitless time of 1 min increased yielded areas by almost 40% compared with the use of a continuous septum purge and shorter splitless times. The injection volume itself can be increased up to 600 µL showing consistent AreaAll yields but this has been shown to decrease the stability of measured values for multiple injections from a single vial.

After adjustment of the purge and trap software to enable trapping of sampled air from multiple vials we had to change the trap filling from Tenax GR mesh 60/80 to HayeSep D mesh 80/100. Measurement of the δ^13^C value of CH_4_ and quantification of N_2_O can be performed with Tenax GR, but cooling to -175°C was mandatory. Nevertheless, quantification of CH_4_ and measurement of the δ^15^N value of N_2_O did not work with Tenax GR. If only analysis of CH_4_ is of interest, the temperature of the HayeSep D trap can be increased to -118°C to -130°C ^2,3^ or even to the higher temperatures reported from distinct adsorption experiments ^4^. In any case it is reported that N_2_O analysis works best at temperatures below -150°C ^5^.

The purge and trap autosampler is originally designed for volatile organic carbon analysis of water samples and therefore uses two G13 side bore needles of different length for purging and these cannot be used with grey-butyl rubber septa and crimped vials. That is why we had to build a double needle which can be used with our vial specifications. First we tried to copy the existing needle arrangement decreasing the needle gauge to 25, but this could neither provide sufficient fixation with Graphite/Vespel® ferrules resisting penetration shear forces, nor protect needles from bending. Using SilTite™ ferrules or perhaps brass ferrules does protect needles from shifting as does using needle gauges of 19 or above.

Without having a second cryogenic trap, separation of N_2_ from CH_4_ was not working with a gas chromatograph starting at a minimum temperature of 35°C. First, we tried to trap CH_4_ with the regular Cold Trap Option of the GC-Isolink I, trapping CO_2_ produced from the CH_4_ by combustion for a few seconds. Chromatographic separation of CH_4_ and N_2_O using a 30 m Poraplot Q opens a window of approx. 100 seconds which was not long enough to separate N_2_ from CO_2_, switch to the N_2_O gas configuration and then measure the peak of N_2_O. That is why separation of N_2_ and CH_4_ is recommend in front of the GC column. As both CH_4_ and N_2_O are trapped before the GC inlet, the time needed to separate CH_4_ from N_2_ is arbitrary. When it comes to trapping, especially of CH_4_ inside a capillary tube, we do not recommend using deactivated fused silica capillary, because retention inside a piece of coated or packed capillary was much more effective. That is why we chose a piece of the same capillary as used inside the gas chromatograph.


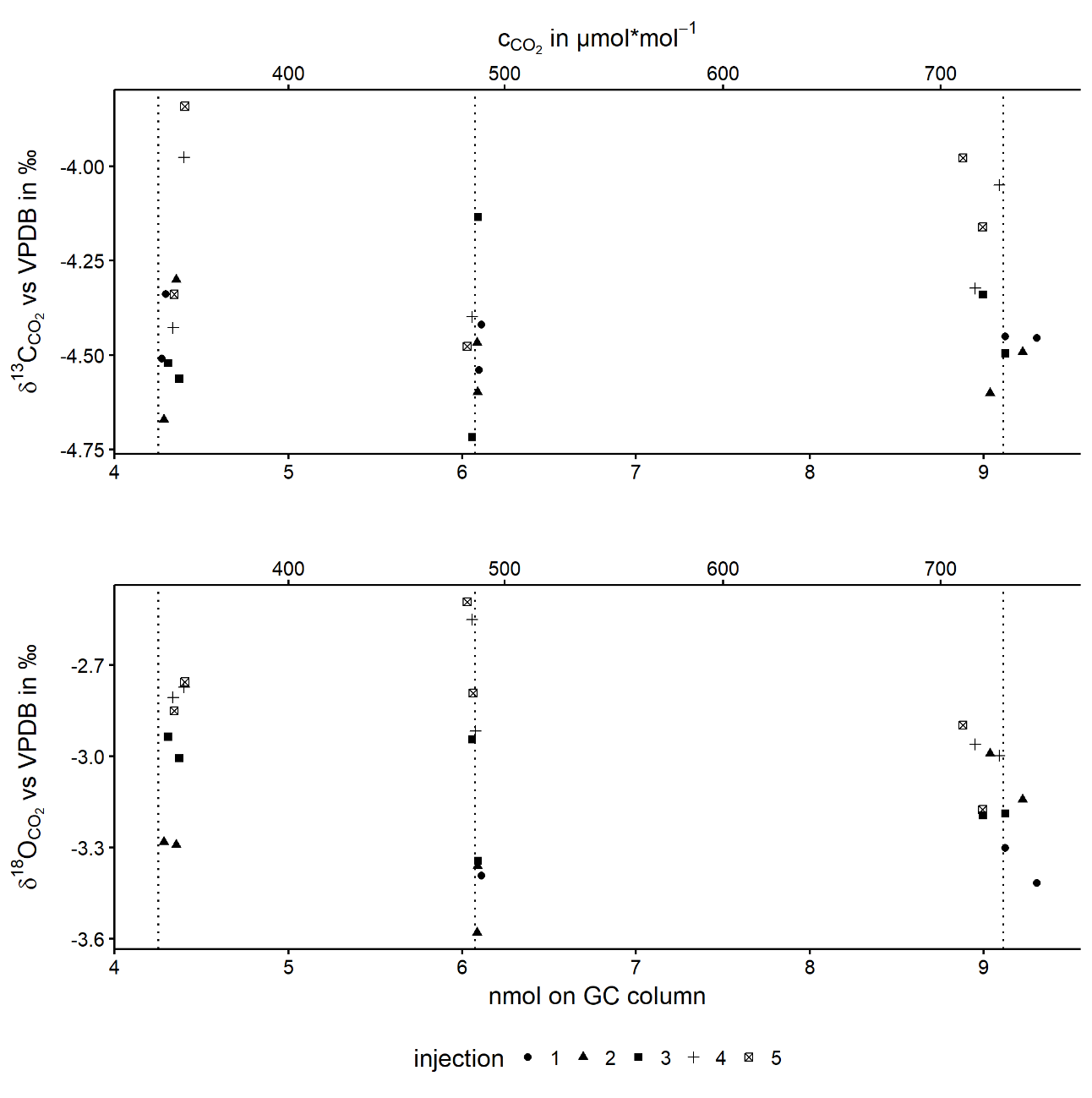


Figure S1: Data points from CO2 working gas standard measurements. Symbols indicate the injection from each vial. Working gas standards were prepared in duplicate in three nominal concentrations (indicated by dotted vertical lines).


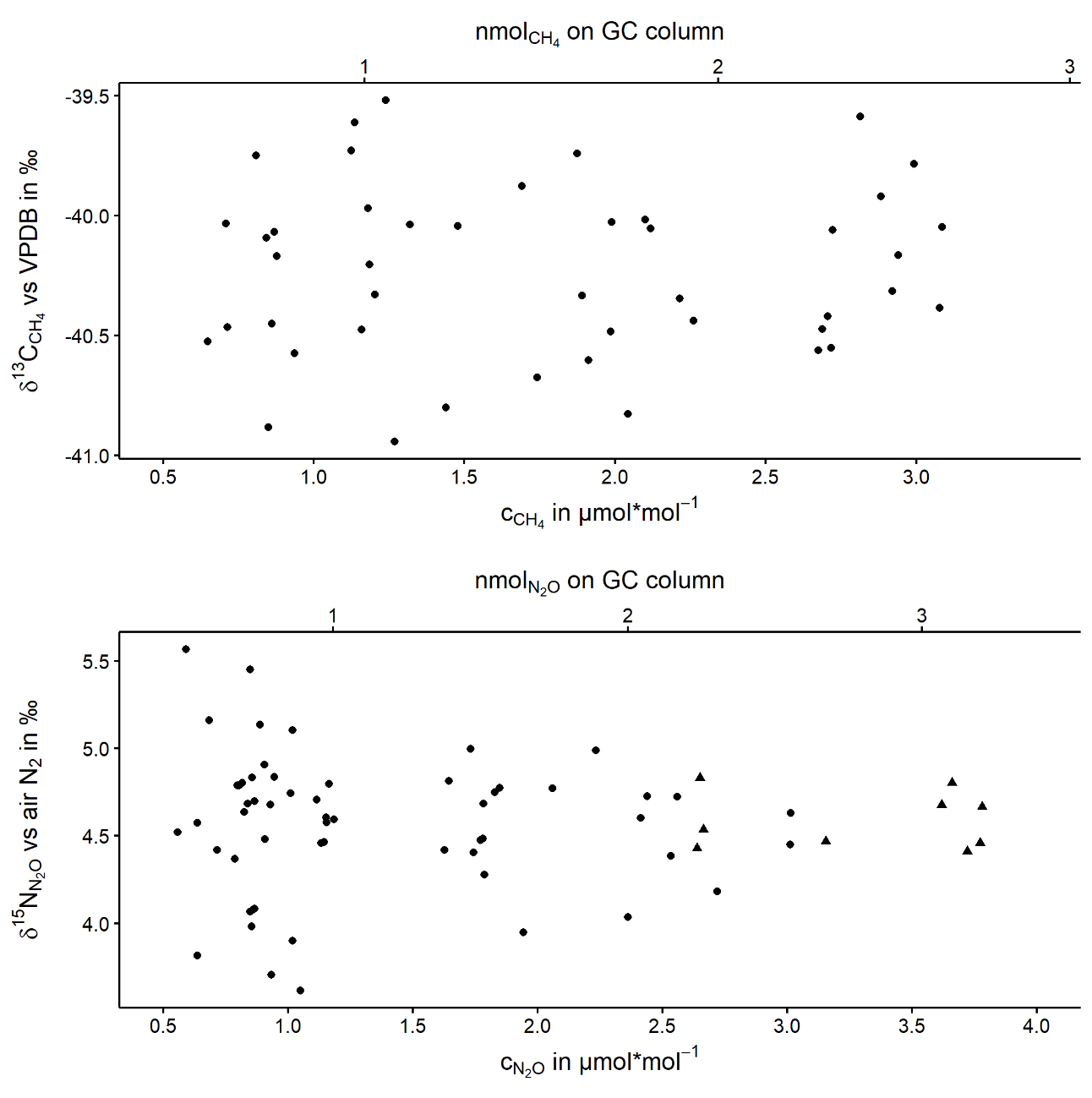


Figure S2: Internal calibration of CH_4_ and N_2_O working gas, dots and triangles represent sample volumes of 20.5 and 61.5 mL, respectively.

**References**

1. McIlvin MR, Casciotti KL. Fully automated system for stable isotopic analyses of dissolved nitrous oxide at natural abundance levels. *Limnol Oceanogr Methods*. 2010;8(2):54-66. doi:10.4319/lom.2010.8.54

2. Merritt DA, Hayes JM, Des Marais DJ. Carbon isotopic analysis of atmospheric methane by isotope-ratio- monitoring gas chromatography-mass spectrometry. *J Geophys Res*. 1995;100(D1):1317-1326. doi:10.1029/94JD02689

3. Rice AL, Gotoh AA, Ajie HO, Tyler SC. High-Precision Continuous-Flow Measurement of δ^13^C and δD of Atmospheric CH_4_. *Anal Chem.* 2001;73(17):4104-4110. doi:10.1021/ac0155106

4. Eyer S, Stadie NP, Borgschulte A, Emmenegger L, Mohn J. Methane preconcentration by adsorption: a methodology for materials and conditions selection. *Adsorption*. 2014;20(5-6):657-666. doi:10.1007/s10450-014-9609-9

5. Mohn J, Guggenheim C, Tuzson B, et al. A liquid nitrogen-free preconcentration unit for measurements of ambient N 2O isotopomers by QCLAS. *Atmos Meas Tech*. 2010;3(3):609-618. doi:10.5194/amt-3-609-2010

6. Tschickardt M. Dinitrogen oxide (nitrous oxide) [Air Monitoring Methods, 2007b]. In: *The MAK-Collection for Occupational Health and Safety*. Weinheim, Germany: Wiley-VCH Verlag GmbH & Co. KGaA; 2012:132-143. doi:10.1002/3527600418.am1002497e0010b
